# Clinical outcome of out-of-hospital vs. in-hospital cardiac arrest survivors presenting with ventricular tachyarrhythmias

**DOI:** 10.1007/s00380-021-01976-y

**Published:** 2021-11-16

**Authors:** Julian Müller, Michael Behnes, Tobias Schupp, Linda Reiser, Gabriel Taton, Thomas Reichelt, Dominik Ellguth, Martin Borggrefe, Niko Engelke, Armin Bollow, Seung-Hyun Kim, Kathrin Weidner, Uzair Ansari, Kambis Mashayekhi, Muharrem Akin, Philipp Halbfass, Dirk Große Meininghaus, Ibrahim Akin, Jonas Rusnak

**Affiliations:** 1 First Department of Medicine, Faculty of Medicine Mannheim, University Medical Centre Mannheim (UMM), European Centre for AngioSience (ECAS) and German Center for Cardiovascular Research (DZHK) Partner Site Heidelberg/Mannheim, Theodor-Kutzer-Ufer 1-3, 68167 Mannheim, Germany; 2 Department of Cardiology and Angiology II, University Heart Center Freiburg, Bad Krozingen, Germany; 3 Department of Cardiology and Angiology, Hannover Medical School, Hannover, Germany; 4 Department of Interventional Electrophysiology, Heart Centre Bad, Neustadt, Germany; 5 Department of Cardiology, Carl-Thiem-Klinikum Cottbus, Cottbus, Germany

**Keywords:** out-of-hospital cardiac arrest, in-hospital cardiac arrest, Ventricular tachyarrhythmias, Cardiopulmonary resuscitation, Cardiac arrest, Ventricular tachycardia, Ventricular fibrillation, Mortality

## Abstract

Limited data regarding the prognostic impact of ventricular tachyarrhythmias related to out-of-hospital (OHCA) compared to in-hospital cardiac arrest (IHCA) is available. A large retrospective single-center observational registry with all patients admitted due to ventricular tachyarrhythmias was used including all consecutive patients with ventricular tachycardia (VT) and fibrillation (VF) on admission from 2002 to 2016. Survivors discharged after OHCA were compared to those after IHCA using multivariable Cox regression models and propensity-score matching for evaluation of the primary endpoint of long-term all-cause mortality at 2.5 years. Secondary endpoints were all-cause mortality at 6 months and cardiac rehospitalization at 2.5 years. From 2.422 consecutive patients with ventricular tachyarrhythmias, a total of 524 patients survived cardiac arrest and were discharged from hospital (OHCA 62%; IHCA 38%). In about 50% of all cases, acute myocardial infarction was the underlying disease leading to ventricular tachyarrhythmias with consecutive aborted cardiac arrest. Survivors of IHCA were associated with increased long-term all-cause mortality compared to OHCA even after multivariable adjustment (28% vs. 16%; log rank *p* = 0.001; HR 1.623; 95% CI 1.002–2.629; *p* = 0.049) and after propensity-score matching (28% vs. 19%; log rank *p* = 0.045). Rates of cardiac rehospitalization rates at 2.5 years were equally distributed between OHCA and IHCA survivors. In patients presenting with ventricular tachyarrhythmias, survivors of IHCA were associated with increased risk for all-cause mortality at 2.5 years compared to OHCA survivors.

## Introduction

Sudden cardiac arrest is common and one of the leading causes of death in developed industrial countries [1]. Most non-traumatic OHCA result from acute coronary syndromes (ACS), structural heart disease or cardiac arrhythmias [1, 2]. In-hospital cardiac arrest (IHCA) data are scarce; however, reported incidences range from 1 to 5 events per 1000 hospital admissions [3, 4]. Immediate cardiopulmonary resuscitation (CPR) and treatment of the underlying cardiac or non-cardiac pathology are necessary for a return of a spontaneous circulation (ROSC).

Mortality rates of cardiac arrest remained stable in the last decades. In Europe, overall survival rate is 10.3% after OHCA and 23% after IHCA [1, 5]. The majority of deaths occur during the initial resuscitation. However, a substantial portion of deaths from cardiac arrest occur in patients with primarily successful resuscitation [6]. This increased mortality post resuscitation can be attributed to a combination of whole-body ischemia, reperfusion-mediated tissue damage and underlying diseases that lead to cardiac arrest [7].

Survival and outcome analyses of OHCA and IHCA can be numerously found in current literature, showing better short-term survival rates for IHCA in many studies [8, 9]. The present study evaluates the long-term prognostic impact of OHCA and IHCA in consecutive patients presenting with ventricular tachyarrhythmias.

## Methods

### Study patients, design and data collection

The present study is derived from an observational analysis of the “Registry of Malignant Arrhythmias and Sudden Cardiac Death—Influence of Diagnostics and Interventions (RACE-IT)” and represents a single-center registry including consecutive patients presenting with ventricular tachyarrhythmias and aborted sudden cardiac death (SCD) being admitted to the University Medical Center Mannheim (UMM), Germany (clinicaltrials.gov identifier: NCT02982473) from 2002 until 2016. The UMM is a large tertiary care University hospital in the metropolitan area of Rhine/Main with over 1.350 beds with optional supply of extracorporeal membrane oxygenation (ECMO) and target temperature management (TTM).

Ventricular tachyarrhythmias comprised ventricular tachycardia (VT) and fibrillation (VF), as defined by current international guidelines [10]. Sustained VT was defined by a duration of > 30 s or causing hemodynamic collapse within 30 s [10].

The overall presence of an activated ICD comprises the total sum of all patients with a prior implanted ICD before admission, those undergoing new ICD implantation at index stay, as well as those with ICD implantation at the complete follow-up period after index hospitalization, referring to transvenous ICD, subcutaneous-ICD (s-ICD) and cardiac resynchronization therapy with defibrillator function (CRT-D). Pharmacological treatment was documented according to the discharge medication of patients surviving index hospitalization.

Values of left ventricular ejection fraction (LVEF) were retrieved from standardized transthoracic echocardiographic examinations usually being performed before hospital discharge in survivors to assess realistic LVEF values beyond the acute phase of ventricular tachyarrhythmias or related acute coronary ischemia. In minor part and only if available, earlier LVEF values assessed on admission or during intensive care were retrieved from patients.

Every re-visit at the outpatient clinic or rehospitalization was documented when related to recurrent ventricular tachyarrhythmias and adverse cardiac events. Adverse cardiac events comprised acute heart failure (AHF), CPR, cardiac surgery, recurrent percutaneous coronary intervention (re-PCI), new implants or upgrades of cardiac devices, worsening or improvement of LVEF.

Documentation period lasted from index event until 2016. Documentation of all medical data was performed by independent cardiologists at the time of the patients´ individual period of clinical presentation, being blinded to final data analyses.

The present study was carried out according to the principles of the declaration of Helsinki and was approved by the medical ethics committee II of the Medical Faculty Mannheim, University of Heidelberg, Germany.

### Definition of study groups, inclusion and exclusion criteria

For the present analysis, risk stratification was performed according to the presence of IHCA and OHCA as defined by current European and American guidelines [10–12]. OHCA group comprises patients with out of hospital ventricular tachyarrhythmias at index hospitalization, who required defibrillation or cardioversion either with or without CPR, or patients with out of hospital ventricular tachyarrhythmias and consecutive asystole requiring CPR. IHCA patients comprise hospitalized patients who developed ventricular tachyarrhythmias, which required defibrillation or cardioversion with or without CPR, or hospitalized patients who developed ventricular tachyarrhythmias and consecutive asystole requiring CPR. IHCA further comprised patients with hemodynamically stable VT on admission requiring CPR due to recurrent ventricular tachyarrhythmias during index hospitalization. Patients were allocated to the IHCA group if cardiac arrest due to ventricular tachyarrhythmias occurred after handing over from emergency physician to hospital staff.

Regarding exclusion criteria, patients without complete follow-up data regarding mortality and those not requiring CPR were excluded. In addition, patients who were not transferred to the university hospital after a cardiac arrest and patients with death during index hospitalization were excluded.

### Study endpoints

The primary endpoint was all-cause mortality at 2.5 years. The overall follow-up period lasted until 2016. All-cause mortality was documented using our electronic hospital information system and by directly contacting state resident registration offices (“bureau of mortality statistics”) across Germany. Secondary endpoints were all-cause mortality at 6 months and cardiac rehospitalization within 2.5 years. Cardiac rehospitalization comprised rehospitalization due to VT, VF, AHF, acute myocardial infarction (AMI) or inappropriate ICD shock.

### Statistical methods

Quantitative data are presented as mean ± standard error of mean (SEM), median and interquartile range (IQR), and ranges depending on the distribution of the data and were compared using the Student’s *t* test for normally distributed data or the Mann–Whitney *U* test for nonparametric data. Deviations from a Gaussian distribution were tested by the Kolmogorov–Smirnov test. Spearman’s rank correlation for nonparametric data was used to test univariate correlations. Qualitative data are presented as absolute and relative frequencies and compared using the Chi^2^ test or the Fisher’s exact test, as appropriate.

First, data of consecutive patients upon admission is given for the entire unmatched cohort in order to present the real-life character of health-care supply at our institution between 2002 and 2016. Here, multivariable Cox regression models were applied for the evaluation of the primary prognostic endpoint within the total study cohort for IHCA vs. OHCA. Multivariable Cox regression models were adjusted for the following clinically relevant covariables: age, sex, diabetes, chronic kidney disease (CKD) (glomerular filtration rate < 60 mL/min per 1.73 m^2^), coronary artery disease (CAD), prior HF, prior AMI, presence of AMI, left ventricular ejection fraction (LVEF) < 35% and overall ICD. Multivariable Cox regression was applied in the entire study group as well as in both subgroups of OHCA and IHCA survivors.

Second, propensity score matching was applied using data from the entire patient cohort. We used 1:1 propensity-scores for OHCA vs. IHCA to assemble matched and well-balanced subgroups. One-to-one ratio for propensity score matching was performed, including survivors of cardiac arrest and applying a non-parsimonious multivariable logistic regression model [13, 14].

Propensity scores were created according to the presence of the following independent variables: age in decades, sex, diabetes mellitus, CAD, AMI at index hospitalization, LVEF, CKD, cardiogenic shock and overall presence of ICD. Uni-variable stratification was performed using the Kaplan–Meier method with comparisons between groups using uni-variable hazard ratios (HR) given together with 95% confidence intervals.

Follow-up periods for evaluation of long-term all-cause mortality were set at 2.5 years according to the median survival time of the entire study cohort to guarantee complete follow-up of at least 50% of patients. Patients not meeting long-term follow-up were censored.

The result of a statistical test was considered significant for *p* < 0.05, *p* values ≤ 0.1 were defined as a statistical trend. SAS, release 9.4 (SAS Institute Inc., Cary, NC, USA) and SPSS (Version 25, IBM Armonk, New York, USA) were used for statistics.

## Results

### Study population

**Fig. 1:**
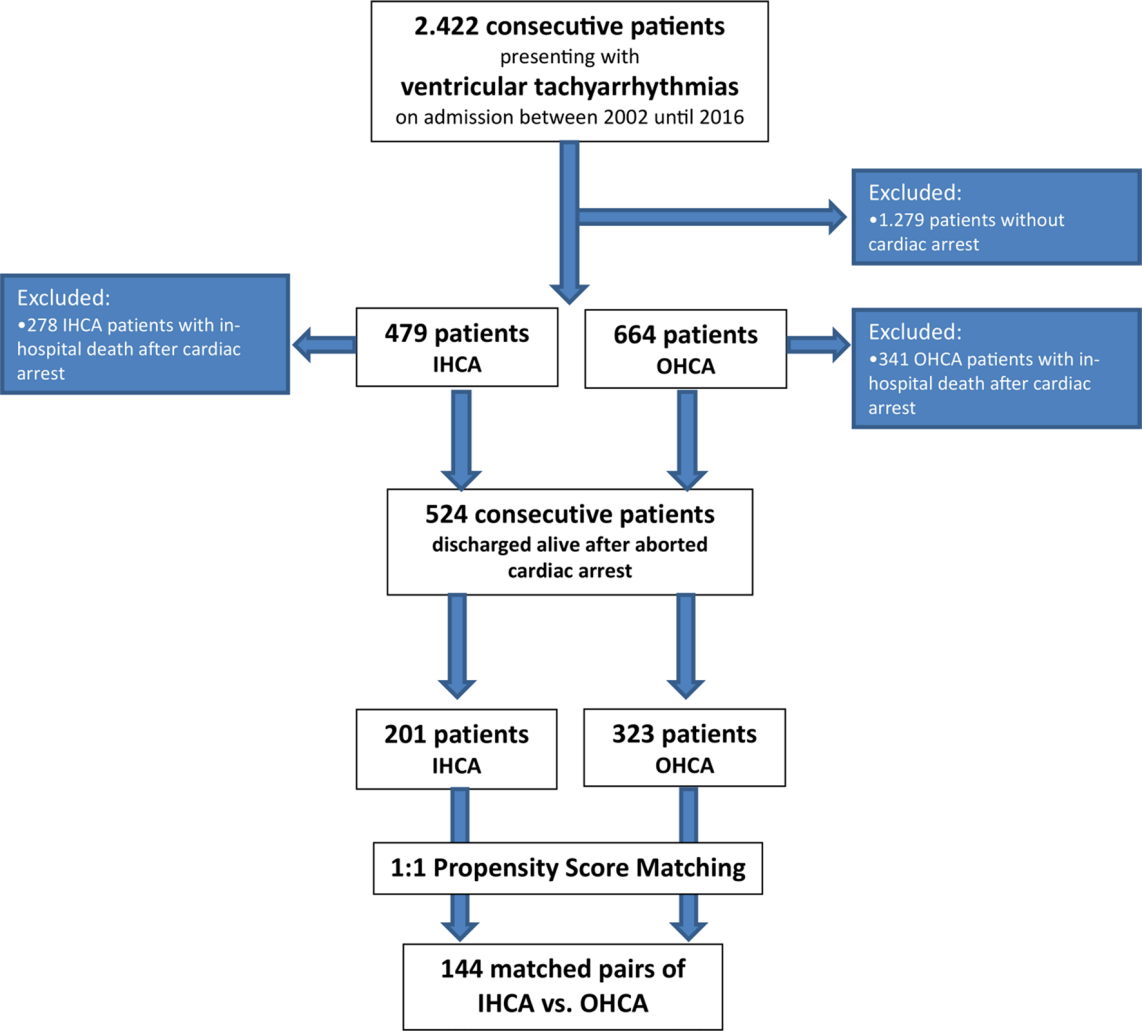
Flow chart of the study population

The present study retrospectively included 2.422 consecutive patients presenting with ventricular tachyarrhythmias from 2002 until 2016 (Fig. 1, flow chart). 1.279 patients were excluded without cardiac arrest, 341 OHCA and 278 IHCA patients died within index hospitalization after cardiac arrest and were, therefore, excluded for the present analysis (Fig. 1, flow chart). Lost to follow-up rate was 1.7% (*n* = 48) regarding survival until the end of the follow-up period.

A total of 524 patients with ventricular tachyarrhythmias and surviving cardiac arrest while being discharged from index hospitalisation were included in this study. 62% of these patients suffered from OHCA. Most patients were males in both subgroups (Table 1, left column). IHCA patients were older, had higher rates of VF compared to VT, as well as higher rates of arterial hypertension and diabetes mellitus. Rates of prior HF, prior CAD, prior AMI, chronic obstructive pulmonary disease (COPD), atrial fibrillation and stroke were higher among IHCA compared to OHCA survivors (Table 1, left column).

**Table 1:** Study cohort

Characteristic	Before matching (*n* = 524)			After matching (*n* = 288)		
	OHCA (*n* = 323; 62%)	IHCA (*n* = 201; 38%)	*p* value	OHCA (*n* = 144; 50%)	IHCA (*n* = 144; 50%)	*p* value
Gender, *n* (%)						
Male	232 (72)	123 (61)	**0.011**	97 (67)	94 (65)	0.708
Age, median (range)	61 (14–89)	68 (20–92)	**0.001**	63 (18–89)	67 (20–90)	0.200
Ventricular tachyarrhythmias, *n* (%)						
Ventricular tachycardia	23 (7)	45 (22)	**0.001**	12 (8)	29 (20)	**0.004**
Ventricular fibrillation	300 (93)	156 (78)		132 (92)	115 (80)	
Cardiovascular risk factors, *n* (%)						
Arterial hypertension	170 (53)	130 (65)	**0.007**	85 (59)	89 (62)	0.630
Diabetes mellitus	71 (22)	72 (36)	**0.001**	39 (27)	43 (30)	0.601
Hyperlipidemia	82 (25)	66 (33)	0.066	38 (26)	51 (35)	0.097
Smoking	111 (34)	66 (33)	0.719	46 (32)	53 (37)	0.385
Cardiac family history	29 (9)	25 (12)	0.205	10 (7)	18 (13)	0.112
Comorbidities, *n* (%)						
Prior heart failure	38 (12)	41 (20)	**0.007**	26 (18)	34 (24)	0.246
Prior coronary artery disease	80 (25)	83 (41)	**0.001**	40 (28)	63 (44)	**0.005**
Prior myocardial infarction	39 (12)	39 (19)	**0.022**	20 (14)	31 (22)	0.090
Chronic kidney disease	172 (54)	82 (41)	**0.005**	60 (42)	59 (41)	0.905
Liver cirrhosis	2 (0.6)	1 (0.5)	1.000	0 (0)	1 (0.7)	0.316
COPD	12 (4)	19 (10)	**0.007**	7 (5)	13 (9)	0.164
Septic shock	1 (0)	7 (3)	**0.006**	1 (1)	7 (5)	**0.018**
Acute myocardial infarction	194 (60)	76 (38)	**0.001**	71 (49)	70 (49)	0.906
Atrial fibrillation	76 (24)	63 (31)	**0.049**	37 (26)	48 (33)	0.155
Stroke	6 (2)	11 (6)	**0.023**	2 (1)	7 (5)	0.090
Intracranial hemorrhage	0 (0)	2 (1)	0.147	0 (0)	0 (0)	–
Coronary artery disease, *n* (%)						
Coronary angiography, overall	273 (85)	147 (73)	**0.001**	122 (85)	112 (78)	0.131
Coronary artery disease, *n* (%)						
No evidence of CAD	46 (17)	34 (23)	0.304	19 (16)	22 (20)	0.877
1-vessel	83 (30)	39 (27)		38 (31)	34 (30)	
2-vessel	69 (25)	30 (20)		28 (23)	24 (21)	
3-vessel	75 (28)	44 (30)		37 (30)	32 (29)	
CABG	22 (8)	13 (9)	0.781	14 (12)	11 (10)	0.682
PCI	182 (67)	77 (52)	**0.004**	72 (59)	66 (59)	0.989
Left ventricular ejection function, *n* (%)						
LVEF ≥ 55%	97 (37)	66 (43)	0.261	58 (40)	60 (42)	0.630
LVEF 54–45%	50 (19)	29 (19)		24 (17)	28 (19)	
LVEF 44–35%	58 (22)	22 (14)		28 (19)	20 (14)	
LVEF < 35%	60 (23)	37 (24)		34 (24)	36 (25)	
Not documented	58 (–)	47 (–)		–	–	
ICD after discharge, *n* (%)	132 (41)	62 (31)	**0.021**	54 (38)	51 (35)	0.713
Primary prevention	22 (7)	22 (11)	0.097	20 (14)	19 (13)	0.863
Secondary prevention	110 (34)	40 (20)	**0.001**	42 (29)	38 (26)	0.599
Medication at discharge, *n* (%)						
Beta-blocker	267 (83)	145 (72)	**0.004**	115 (80)	112 (78)	0.665
ACE inhibitor	217 (67)	129 (64)	0.480	93 (65)	100 (69)	0.380
ARB	17 (5)	18 (9)	0.090	6 (4)	11 (8)	0.195
Digitalis	20 (6)	21 (10)	0.078	12 (8)	17 (12)	0.328
Amiodarone	27 (8)	31 (15)	**0.012**	15 (10)	19 (13)	0.465

Bold values mean *p* value is significant (< 0.05)

*ACE* angiotensin converting enzyme, *ARB* angiotensin receptor blocker, *CABG* coronary artery bypass grafting, *CAD* coronary artery disease, *COPD* chronic obstructive pulmonary disease, *CRT-D* cardiac resynchronisation therapy with defibrillator, *ICD* implantable cardioverter–defibrillator, *IHCA* in-hospital cardiac arrest, *LVEF* left ventricular ejection fraction, *OHCA* out of hospital cardiac arrest, *PCI* percutaneous coronary intervention

In OHCA patients, coronary angiography and subsequent PCI was performed more often. Higher rates of ICD implantation after cardiac arrest were present in OHCA survivors, mainly due to secondary preventative ICDs (Table 1, left column).

### Associated diseases for cardiac arrest

**Fig. 2:**
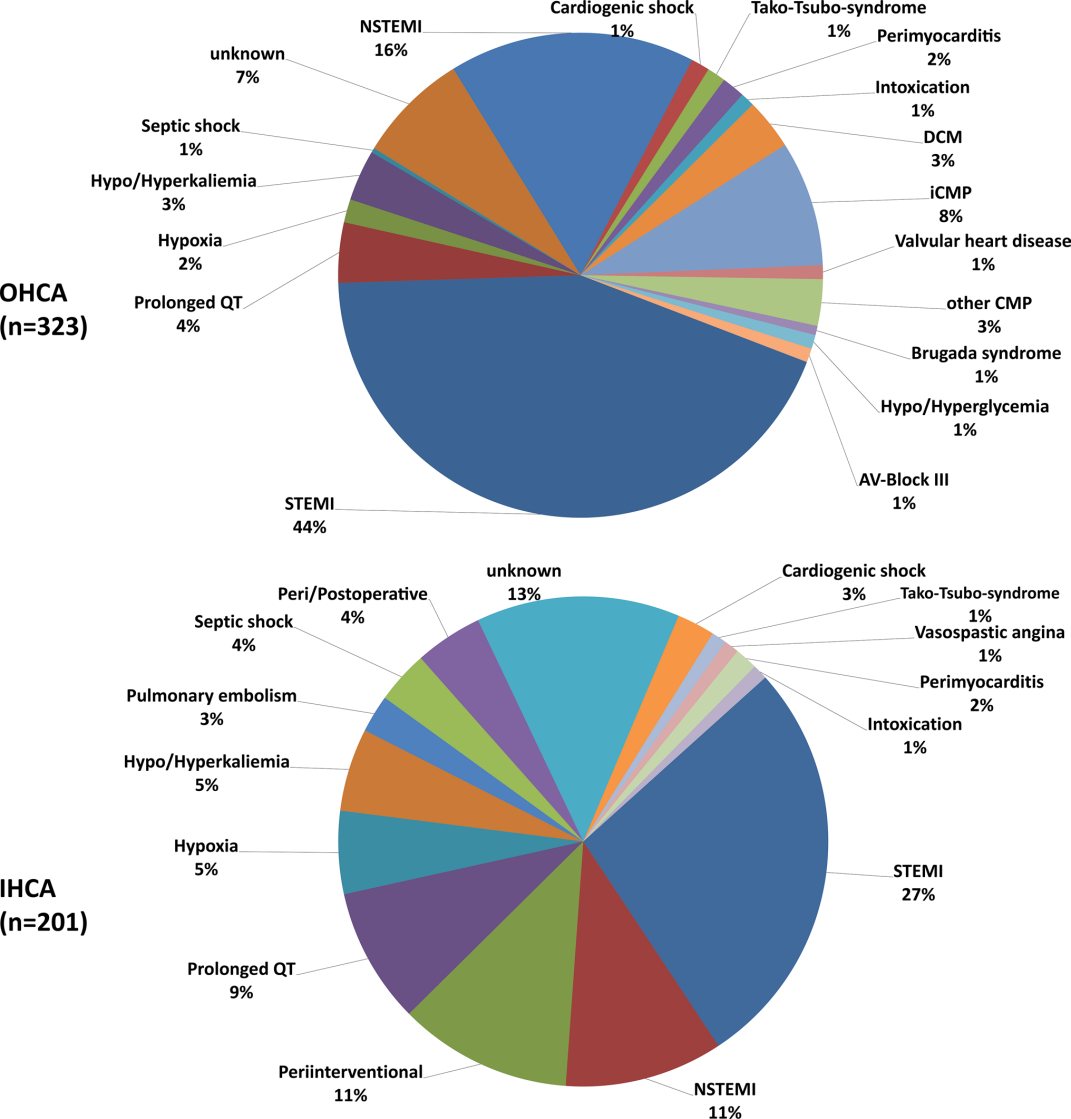
Associated diseases for cardiac arrest. *AV block* atrioventricular block, *CMP* cardiomyopathy, *DCM* dilative cardiomyopathy, *iCMP* ischemic cardiomyopathy, *IHCA* in-hospital cardiac arrest, *NSTEMI* non-ST-segment-elevation myocardial infarction, *OHCA* out-of-hospital cardiac arrest, *STEMI* ST-segment-elevation myocardial infarction

Most cardiac arrests resulted from myocardial infarction (52%). Among OHCA survivors, higher rates of STEMI (44% vs. 27%; *p* = 0.001) as well as NSTEMI (16% vs. 10%; *p* = 0.070) were common. Furthermore, higher rates of cardiomyopathies, such as ischemic cardiomyopathy (8% vs. 0%; *p* = 0.001), DCM (3% vs. 0%; *p* = 0.009) and other rare cardiomyopathies (3% vs. 0%; *p* = 0.009) as associated diseases were present among OHCA survivors. IHCA survivors suffered more often from prolonged QT intervals (9% vs. 4%; *p* = 0.023), respiratory failure with consecutive hypoxia (5% vs. 2%; *p* = 0.017), septic shock (3% vs. 0%; *p* = 0.006) and pulmonary embolism (2% vs. 0%; *p* = 0.008). A significant amount of IHCA was associated with peri-interventional VF during coronary angiography (11% vs. 0%; *p* = 0.001) or operations (4% vs. 0%; *p* = 0.001). In 10% of all cases no associated reason for cardiac arrest was found (Table 2; Fig. 2).

**Table 2:** Associated diseases in overall study cohort

Characteristic	OHCA (*n* = 323; 62%)	IHCA (*n* = 201; 38%)	*p* value
Cardiac, *n* (%)			
STEMI	141 (44)	55 (27)	**0.001**
NSTEMI	53 (16)	21 (10)	0.070
Cardiogenic shock	4 (1)	5 (2)	0.314
Tako-Tsubo-CMP	4 (1)	2 (1)	1.000
Perimyocarditis	5 (2)	3 (1)	1.000
DCM	11 (3)	0 (0)	**0.009**
iCMP	27 (8)	0 (0)	**0.001**
Valvular heart disease	3 (1)	0 (0)	0.289
Other CMP	10 (3)	0 (0)	**0.009**
Brugada syndrome	2 (1)	0 (0)	0.526
Prolonged QT	13 (4)	18 (9)	**0.023**
Third-degree AV Block	3 (1)	0 (0)	0.289
Vasospastic angina	–	2 (1)	0.147
Pulmonary embolism	–	5 (2)	**0.008**
Pulmonary, *n* (%)			
Hypoxia	5 (2)	11 (5)	**0.017**
Other, *n* (%)			
Hypo/Hyperkaliemia	11 (3)	11 (5)	0.269
Septic shock	1 (0)	7 (3)	**0.006**
Intoxication	3 (1)	2 (1)	1.000
Hypo/Hyperglycemia	3 (1)	–	0.289
Periinterventional	–	23 (11)	**0.001**
Peri/postoperative	–	9 (4)	**0.001**
Unknown	24 (7)	27 (13)	**0.033**

Bold values mean *p* value is significant (< 0.05)

*AV block* atrioventricular block, *CMP* cardiomyopathy, *DCM* dilative cardiomyopathy, *iCMP* ischemic cardiomyopathy, *IHCA* in-hospital cardiac arrest, *NSTEMI* non-ST-segment-elevation myocardial infarction, *OHCA* out-of-hospital cardiac arrest, *STEMI* ST-segment-elevation myocardial infarction

**Fig. 3:**
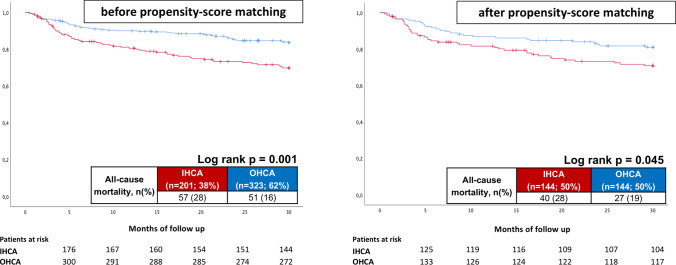
IHCA was associated with increased long-term all-cause mortality at 2.5 years compared to OHCA before (left panel) and after propensity score matching (right panel)

### Characteristics related to resuscitation

In most patients, initial rhythm was VF (93% vs. 78%; *p* = 0.001), which was treated with defibrillator in most cases (91% vs. 73%; *p* = 0.001). Median time from initiation of CPR to ROSC was higher among OHCA patients, whereas TTM was performed more frequently in patients with OHCA. Patients with IHCA were cardioverted more often (Table 3**)**.

**Table 3:** Characteristics related to CA

Characteristic	OHCA (*n* = 323; 62%)	IHCA (*n* = 201; 38%)	*p* value
Initial rhythm, *n* (%)			
Ventricular tachycardia	23 (7)	45 (22)	**0.001**
Ventricular fibrillation	300 (93)	156 (78)	
Time from CPR to ROSC, min, median (IQR)	15 (4–32)	5 (2–12)	1.000
Defibrillation, *n* (%)	294 (91)	146 (73)	**0.001**
Number of shocks, *n* (%)			
1	116 (36)	101 (50)	**0.001**
2	59 (18)	21 (11)	
≥ 3	119 (37)	24 (12)	
Thrombolysis, *n* (%)	17 (5)	9 (5)	0.687
Cardioversion	6 (2)	12 (6)	**0.012**
Targeted temperature management	77 (24)	3 (2)	**0.001**

Bold values mean *p* value is significant (< 0.05)

*CA* cardiac arrest, *CPR* cardiopulmonary resuscitation, *IHCA* in-hospital cardiac arrest, *IQR* interquartile range, *OHCA* out-of-hospital cardiac arrest, *ROSC* return of spontaneous circulation

### Primary and secondary endpoints

Median follow-up time was 6.3 years for OHCA survivors and 4.8 years for IHCA survivors (IQR 1616–1221 days). As shown in Table 4, left columns, IHCA survivors were associated with higher rates of all-cause mortality at 2.5 years compared to OHCA survivors (28% vs. 16%, log rank *p* = 0.001; HR 2.005; CI 95% 1.374–2.926; *p* = 0.001) (Fig. 3, left). IHCA survivors were also associated with higher rates of the secondary endpoint all-cause mortality at 6 months (14% vs. 7%, log rank *p* = 0.010). Cardiac rehospitalization rates at 2.5 years were equally distributed between OHCA and IHCA survivors.

**Table 4:** Primary and secondary endpoints

Characteristics	Before matching (*n* = 524)			After matching (*n* = 288)		
	OHCA (*n* = 323; 62%)	IHCA (*n* = 201; 38%)	*p* value	OHCA (*n* = 144; 50%)	IHCA (*n* = 144; 50%)	*p* value
Primary endpoint, *n* (%)						
All-cause mortality, at 2.5 years	51 (16)	57 (28)	**0.001**	27 (19)	40 (28)	**0.045**
Secondary endpoints, *n* (%)						
All-cause mortality, at 6 months	24 (7)	29 (14)	**0.010**	12 (8)	22 (15)	0.068
Cardiac rehospitalization, at 2.5 years	30 (9)	23 (11)	0.426	7 (5)	15 (10)	0.076
Follow up times, *n* (%)						
Hospitalization total; days (median (IQR))	18 (11–31)	20 (11–34)	0.069	17 (10–30)	19 (11–33)	0.157
ICU time; days (median (IQR))	9 (1–14)	5 (3–12)	0.123	8 (3–13)	5 (3–11)	0.822
Follow-up; days (mean; median (range))	1758; 1616 (3–4542)	1537; 1221 (15–4724)	**0.049**	1890; 1816 (25–4542)	1621; 1287 (15–4574)	0.075

Bold values mean *p* value is significant (< 0.05)

*ICU* invasive care unit, *IHCA* in-hospital cardiac arrest, *IQR* interquartile range, *OHCA* out of hospital cardiac arrest

### Multivariable Cox regression models

After applying a multivariable Cox regression model, IHCA was still associated with the primary endpoint of all-cause mortality at 2.5 years compared to OHCA (HR 1.623; 95% CI 1.002–2.629; *p* = 0.049) (Table 5; right panel). Furthermore, age, CKD and impaired LVEF were associated with increased all-cause mortality, whereas AMI and the presence of an activated ICD were associated with improved survival in all survivors of cardiac arrest.

**Table 5:** Unmatched uni- and multivariable hazard ratios to predict the primary prognostic endpoint of long-term all-cause mortality at 2.5 years (*n* = 524)

	Univariable			Multivariable		
	HR	95% CI	*p* value	HR	95% CI	*p* value
Age	1.046	1.030–1.062	**0.001**	1.026	1.006–1.047	**0.009**
Male gender	1.087	0.722–1.638	0.689	1.433	0.869–2.363	0.158
Diabetes	2.265	1.548–3.314	**0.001**	1.301	0.808–2.096	0.279
CKD	2.211	1.481–3.303	**0.001**	1.926	1.177–3.153	**0.009**
CAD	0.980	0.643–1.494	0.926	1.232	0.681–2.229	0.490
Prior HF	2.145	1.388–3.316	**0.001**	1.338	0.789–2.266	0.280
Prior AMI	1.503	0.933–2.421	0.094	1.107	0.621–1.974	0.730
AMI	0.507	0.341–0.752	**0.001**	0.433	0.251–0.747	**0.003**
LVEF	1.351	1.128–1.619	**0.001**	1.301	1.056–1.603	**0.013**
Overall ICD	0.483	0.311–0.751	**0.001**	0.351	0.205–0.603	**0.001**
IHCA (vs. OHCA)	2.005	1.374–2.926	**0.001**	1.623	1.002–2.629	**0.049**

Bold type indicates statistical significance *p* < 0.05

*AMI* acute myocardial infarction, *CAD* coronary artery disease, *CI* confidence interval, *CKD* chronic kidney disease, *HF* heart failure, *HR* hazard ratio, *ICD* implantable cardioverter defibrillator, *IHCA* in-hospital cardiac arrest, *LVEF* left ventricular ejection faction, *OHCA* out of hospital cardiac arrest

As demonstrated in Table 6, age and CKD were significant predictors of all-cause mortality at 2.5 years in OHCA survivors, whereas AMI and an activated ICD were beneficial. In IHCA survivors, only CKD was associated with increased risk of long-term all-cause mortality at 2.5 years.

**Table 6:** Multivariable hazard ratios to predict the primary prognostic endpoint of long-term all-cause mortality at 2.5 years in OHCA compared to IHCA survivors

	OHCA (*n* = 323)			IHCA (*n* = 201)		
	HR	95% CI	*p* value	HR	95% CI	*p* value
Age	1.045	1.013–1.079	**0.005**	1.010	0.984–1.037	0.448
Male gender	1.048	0.490–2.242	0.903	1.916	0.972–3.777	0.060
Diabetes	1.714	0.797–3.684	0.168	0.990	0.515–1.903	0.976
CKD	2.582	1.137–5.865	**0.023**	2.098	1.074–4.097	**0.030**
CAD	1.366	0.460–4.058	0.574	0.891	0.410–1.936	0.771
Prior HF	1.077	0.382–3.039	0.888	1.552	0.762–3.161	0.226
Prior AMI	0.706	0.262–1.902	0.491	1.568	0.730–3.364	0.249
AMI	0.299	0.128–0.700	**0.005**	0.491	0.238–1.016	0.055
LVEF < 35%	1.349	0.962–1.894	0.083	1.107	0.831–1.474	0.489
Overall ICD	0.156	0.066–0.371	**0.001**	0.744	0.364–1.518	0.416

Bold type indicates statistical significance *p* < 0.05

*AMI* acute myocardial infarction, *CAD* coronary artery disease, *CI* confidence interval, *CKD* chronic kidney disease, *HF* heart failure, *HR* hazard ratio, *ICD* implantable cardioverter defibrillator, *IHCA* in-hospital cardiac arrest, *LVEF* left ventricular ejection faction, *OHCA* out of hospital cardiac arrest

### Propensity-matched cohort

After using propensity-score matching for comparison of OHCA and IHCA survivors 288 matched pairs were achieved with similar baseline characteristics (Table 1, right columns).

Figure 3 (right) illustrates the increased risk for long-time all-cause mortality in IHCA patients compared to OHCA patients presenting with ventricular tachyarrhythmias on hospital admission after propensity-score matching (primary endpoint, all-cause mortality at 2.5 years: 28% vs. 19%; log-rank *p* = 0.045). No significant differences regarding secondary endpoints were observed after propensity-score matching (Table 4, right columns)

## Discussion

This study evaluates the prognostic impact of OHCA compared to IHCA in consecutive patients presenting with ventricular tachyarrhythmias and aborted cardiac arrest and survival until hospital discharge from 2002 and 2016 at one institution.

This study suggests that patients with ventricular tachyarrhythmias represent a group with the highest risk for consecutive sudden or aborted cardiac arrest. The present study showed that IHCA is associated with higher long-term all-cause mortality at 6 months and 2.5 years. This reduced long-term survival of IHCA was even present after multivariable adjustment and propensity score matching. Therefore, this study might identify patients hospitalized due to ventricular tachyarrhythmias developing IHCA as a highest risk patient cohort being endangered of worst prognosis. Notably, since most of the cardiac arrest literature is focused on survival to admission or survival to discharge, patients with an inpatient death were excluded.

It is well known that short-term survival is more likely for IHCA patients compared to OHCA patients [8]. This might be due to the professional setting in hospitals with minimal delay from cardiac arrest to cardioversion or defibrillation and availability of diagnostic and therapeutic options, such as imaging, percutaneous coronary intervention and VT ablation [15]. Our analysis suggests that the initial survival benefit of IHCA patients might not persist as IHCA presents adverse long-term survival compared to OHCA. However, it might be speculated that not all OHCA patients had reached the hospital, possibly impacting the lower mortality among OHCA patients.

VT is often caused by a scar-related substrate due to ischemic cardiomyopathy, structural or inflammatory heart disease [10]. This can also be seen in our cohort, where among IHCA survivors greater rates of prior CAD were present and, accordingly, higher rates of VT could be detected. VF usually occurs in acute myocardial ischemia [10]. Accordingly, the present study demonstrated that OHCA survivors suffered more often from VF as substrate of ischemia during AMI, which is also reflected in higher rates of STEMI and NSTEMI as well as overall rates of CAD among OHCA survivors.

In total, cardiac related resuscitation was much more common among OHCA survivors, which is in line with current literature. Most frequent underlying pathology for OHCA is AMI, being responsible for up to 50% of all cases [16]. Interestingly, despite better intra-hospital diagnostic possibilities, IHCA revealed higher rates of unknown possible underlying cause for occurrence of ventricular tachyarrhythmias with resuscitation. Other trials investigating IHCA reported even higher rates of unknown causes for IHCA [17].

Well established predictors of adverse outcome after cardiac arrest are high lactate and high creatinine levels [3]. Accordingly, the strongest negative effect on long-term all-cause mortality in this study was the presence of CKD in both OHCA and IHCA. Recently, it was shown that the presence of CKD and, especially of renal replacement therapy, was associated with increased long-term mortality among patients with ventricular tachyarrhythmias and aborted cardiac arrest [18]. Hence, among IHCA patients lower lactate and creatinine levels were associated with higher likelihood of survival [19]. However, the latter study included only selected patients, in particular patients treated with extracorporeal cardiopulmonary resuscitation (ECPR) and comprised patients with cardiac and non-cardiac etiology of cardiac arrest [19]. Nevertheless, acute kidney injury (AKI) is common among post-resuscitated patients constituting a major risk factor not only for subsequent mortality but also for poor neurological outcome [20]. The connection between AKI and neurological outcome after resuscitation is still not clear, but it could be speculated that both reflect total ischemia time and severe chronic comorbid conditions [21]. In general, presence and severity of AKI could be used as a marker for hypoxia duration during cardiac arrest. For other acute conditions leading to kidney hypoxia, such as sepsis, cardiogenic shock or haemorrhage, this interaction is well investigated [22, 23]. Accordingly, also in this study OHCA patients revealed higher prevalence of kidney disease as a marker of prolonged ischemia time compared to IHCA patients with is also consistent with increased CPR to ROSC time among those patients. However, investigation of detailed values of lactate levels, AKI and renal replacement therapy was beyond the scope of this study.

An ICD remains the most effective treatment for primary and secondary prevention of sudden cardiac death (SCD) [24]. Interestingly, presence of an activated ICD was effective only among OHCA survivors. OHCA patients suffered significantly more often from AMI with consecutive ventricular tachyarrhythmias representing a potentially reversible disease after coronary angiography and revascularization. IHCA patients, on the other hand, suffered more often from hypoxia, sepsis and septic shock as possible underlying reason for need of resuscitation constituting a complex pathology with protracted hospitalization. Furthermore, higher rates of comorbidities such as arterial hypertension, diabetes mellitus, prior HF and CAD as well as lower cardiac interventions such as defibrillation were present among IHCA patients and OHCA survivors presented higher rates of ICD implantation after cardiac arrest. Especially increased rates of pre-existing CAD among IHCA were persistent even after propensity score matching. CAD was counted with evidence of at least one coronary arterial stenosis > 50% in at least one coronary artery or a history of percutaneous coronary intervention (PCI) or coronary artery bypass grafting (CABG). However, it was beyond the scope of the study to investigate the severity and distribution of CAD. Altogether, increased mortality among IHCA patients might be due to increased preexisting conditions compared to OHCA patients. In addition, univariate and multivariate analysis support this hypothesis, since mortality rates in IHCA patients were mainly driven by CKD, but not by previous existing CAD.

This study demonstrates increased long-term all-cause mortality at 2.5 years in IHCA survivors compared to OHCA survivors presenting consecutively with ventricular tachyarrhythmias on admission. Increasing all-cause mortality was already present after 6 months of follow-up. Multivariate Cox regression demonstrated an adverse effect of CKD both in OHCA and IHCA. It might be speculated, whether kidney disease may serve as a marker of hypoxia duration.

In summary, despite better in-hospital survival for IHCA patients compared to OHCA survivors, long-term prognosis of IHCA patients seems to be very poor highlighting the need for a better standardized follow-up therapy of this vulnerable patient cohort. Regarding the higher rates of long-term mortality, we propose a broad diagnostic for IHCA patients in combination with guideline-directed ICD implantation and close follow-up examinations. In addition, diagnostic and treatment of pre-existing non-cardiac conditions should move into cardiologist’s focus. However, this still needs to be reevaluated in future prospective randomized trials.

### Study limitations

This observational and retrospective registry-based single centre analysis reflects a realistic picture of consecutive health-care supply of high-risk patients presenting with ventricular tachyarrhythmias. In addition, heterogeneity within the study population was controlled by a stepwise statistical approach including multivariable adjustment for several important comorbidities and risk factors, both within the entire and propensity matched cohorts. However, some factors among both groups were still present, especially increased rates of previous CAD among IHCA survivors, possibly influencing worse long-term survival, especially because decreased long-term survival was driven by prior AMI (but not by previous CAD). Furthermore, cofounding may still be present due to missing data regarding CPR (i.e., duration until return of spontaneous circulation, pH, neurological status or hypoxia). Only all-cause mortality rates were documented, detailed analyses about definite cause of death were beyond the scope of the study. Among OHCA survivors there is a survival bias, since OHCA patients must have survived both to hospital admission and to discharge without any further episode of ventricular tachyarrhythmia, whereas IHCA survivors “only” had to survive to discharge. Hospital admissions were only documented within our own institution.

## Conclusions

In patients presenting with ventricular tachyarrhythmias, IHCA survivors had a higher risk for all-cause mortality after 6 months and 2.5 years compared to OHCA survivors after discharge from hospital.
